# Virtual tree, real impact: how simulated worlds associate with the perception of limited resources

**DOI:** 10.1057/s41599-022-01225-1

**Published:** 2022-06-24

**Authors:** Manh-Toan Ho, Thanh-Huyen T. Nguyen, Minh-Hoang Nguyen, Viet-Phuong La, Quan-Hoang Vuong

**Affiliations:** 1 Centre for Interdisciplinary Social Research, Phenikaa University, Hanoi, 100803 Vietnam; 2 National Economics University, Hanoi, 100000 Vietnam

**Keywords:** Cultural and media studies, Cultural and media studies, Environmental studies, Science, technology and society

## Abstract

Video games have long been considered an effective educational tool. Environmental education studies have found that games positively affect the feeling of nature connectedness, producing pro-environmental attitudes and behaviors. With growing urbanization, video games also provide chances to interact with nature. During the COVID-19 lockdown, Nintendo’s Animal Crossing: New Horizon (ACNH) became a household name, with millions of copies sold worldwide. The article used the Bayesian multilevel model to analyze 640 survey responses of ACNH game players from various online communities. The correlations between the perception of limited resources and virtual planting and exploiting behaviors with the varying effect among ethnicities were explored. The findings suggested positive correlations between the perception and in-game actions among all ethnicities, regardless of whether the actions are planting or exploiting. While further evidence is needed, the findings suggest the restraints of game mechanics. To foster a pro-environmental culture, stakeholders can consider video games a novel technological aid to environmental education.

## Introduction

Environmental perception of natural resource scarcity has been a concern in the research agenda in economics, public policies, sustainability education, and environmental studies. Mounting evidence has shown that various environmental changes (e.g., biodiversity loss, climate change, and deforestation) are driven by rampant human population growth and unsustainable exploitation activities (Hedges et al. 2018; Steffen et al. 2018). These changes signify that the speed of exploitation has exceeded the Earth’s ability to replenish and support humanity (Becker and Ostrom, 1995). In addition, the increasing scarcity of natural resources places humanity in a dilemma because restoring a degraded environment and its resources may take a long time, or it may never happen naturally even after exploitation is long halted (Folke et al. 2004; Moreno-Mateos et al. 2017; Scheffer and Carpenter, 2003). This challenge urges communities worldwide to promote a more sustainable way of exploiting natural resources, but implementing sustainable practices meets various challenges due to the mixed perceptions of the Earth’s finite resources.

Even though video games were invented in the 1950s, video games have transformed into an increasingly complex combination of the virtual and real world. They provide scientists of various disciplines with a flexible platform and various features to conduct experiments and study players’ minds and behaviors. For example, the Learning Strategies Program, a project initiated in 1976 and funded by Defense Advanced Research Projects Agency, designed a videogame called Space Fortress to observe the learning and skill acquisition process (Donchin, 1989). In the world of video games, resource management is an important mechanic. There are at least 2700 titles with the tag “resource management” on Steam for players to choose from. Most of these games range from simulation genre (like *Cities: Skylines*) to real-time strategy (such as the *Age of Empires* series), or even survival games (like *This War of Mine*). These games provide players with an initial condition and a clear goal (to develop or survive). The limited resource at the initial condition requires players to strategize and find the best way to reach the goal. While resource management is an explicit element in these games’ designs, the mechanism also implicitly exists in other genres such as first-person shooter (FPS) or role-playing games (RPG) (gamemechanics.fandom.com; learn.canvas.net). For instance, managing health and ammunition in shooting games or your avatar’s statistics in an RPG game.

The organic nature of resource management in video games has offered a new educational tool to teach sustainability or natural resource management (Barreteau et al. 2007a, 2007b; Bots and van Daalen, 2007; Ludology, 2020). For instance, BUTORSTAR was specifically designed for raising awareness about reedbed management, accounting for decisions from various stakeholders like farmers, harvesters, or hunters (Mathevet et al. 2007). Similarly, World Climate or Climate Action simulation shares the same goal of providing effective climate change education (Kwok, 2019; Rooney-Varga et al. 2019; Sterman et al. 2015). Recently, game developers have also actively taken part in bringing out video games’ potential in supporting climate action. Notable events such as Climate Jam where game creators make games focusing on climate issues, or initiatives like Game4Sustainability where players can find games based on United Nations’ Sustainable Development Goals, indicate what video games are capable of (Game4Sustainability; indiecade, 2020). Hence, in this article, we explore the association between perception regarding the limits of resources and pro-environmental behaviors by using a sample of 640 Nintendo Animal Crossing: New Horizon (ACNH) players from over 30 countries.

ACNH is a life simulation game released in March 2020 that incorporates economic principles of production, trade, and consumption (Hansen, 2020). The Animal Crossing series, which was first released in 2001, has faced criticisms for its capitalist logic (Bogost, 2008; Vossen, 2020). In the game, players need to extract the island’s resources such as tree products (fruit and wood), fish, and bugs to make money, craft, upgrade the island, and complete tasks. One central economic problem hinders the way: these resources are not abundant. Fruits, for instance, need time to regrow after harvesting, and trees only offer a limited amount of wood for a time. Players can reserve a portion of fruits instead of selling them to grow more trees and conserve trees to guarantee future utilization. However, unlike other genres that heavily focus on resource management like simulation, real-time strategy (RTS), or role-playing games (RPG), ACNH is not entirely about resource management in a constrained scenario. Similar to another simulation game *The Sims*, the island in ACNH is open-ended with options and activities to create the best island life (Griebel, 2006). The problem of limited resources and space depicted in ACNH, while remaining light-hearted, still allows us to examine the association between out-of-game perception of scarcity and in-game pro-environmental behaviors. Moreover, the game’s popularity is also another factor that we decided to choose this game for our study. Within 9.5 months after its release, ACNH’s sales reached 31 million units (Arstechnica.com). The game was also one of the most tweeted about games on Twitter in 2021 (Doolan, 2022). Normally, popular games are often action, first-person shooter (FPS), or racing genres. Thus, even though ACNH is a life simulation with detailed depictions of a virtual environment, the game can still reach a wide range of audiences. Such context provides a unique opportunity.

In the next part, we will review the literature regarding video games and the perception of limited resources. Then, the methodology section provides details about the survey, the dataset, and the Bayesian multilevel model. Finally, we present the results and illustrate how pro-environmental behaviors could be motivated or demotivated by economic and other needs in the game. The article also discusses how game design could motivate pro-environment behaviors and perception.

## Literature review

### The link between exposure to nature, environmental perception, and behavior

Previous works had laid a theoretical foundation for the interactive relationship between perception and behavior (Reibstein et al. 1980). On the one hand, people’s behavior is partially conditioned by their perception of the surrounding environment. For example, social perception directly impacts social behavior, and inputs from the perceptual process would be translated into the corresponding behaviors (Dijksterhuis and Bargh, 2001). On the other hand, the formation of our perception, one of our major sources to acquire knowledge of the external or environmental world, is dependent on what our behaviors allow us to obtain. The organism was argued to have the purpose of its behaviors as a reference point and would navigate what it senses to or near this point (Cziko, 1992; Marken, 2021; Powers, 1973).

Diminishing connection with nature was considered one of the primary problems behind the low perception of environmental crisis and the environmental crisis itself (Maller et al. 2009; Nisbet et al. 2009; Pyle, 2003; Swaisgood and Sheppard, 2011). Therefore, increasing exposure to the environment is proposed as a prerequisite to improving environmental perception, but it met various obstacles, such as the high speed of urbanization (La Puma, 2019; Zylstra et al. 2014). Impoverished interaction with nature among Chinese children was also found to mediate biophobia, which significantly affected their concern towards environmental issues (Zhang et al. 2014). Regarding the relationship between environmental perception and behaviors, it was shown how experience with nature could profoundly influence the way individuals form conceptual knowledge about the external world. In an experiment exploring how exposure to the biological world and cultural backgrounds affects people’s understanding of the biological world, Atran and Medin (2008) found the ways people interact with the world navigate their way of learning and making inferences about nature. Daily interactions with nature and background knowledge also affect individuals’ reasoning patterns when categorizing birds and trees (Atran and Medin, 2008; Medin et al. 1997). For example, while sorting patterns of professional taxonomists and maintenance personnel relying on category-based reasoning, landscape workers categorized trees based on utilitarian concerns (Medin et al. 1997). It implied that how individuals interact with nature can reflect their perception of the external world.

### The controversial link between players’ in-game behavior and their out-of-game perception

Researchers have explored the link between playing video games and the real world’s perception or behavior changes. However, the primary focus has been on video games and violent behaviors for various reasons. Research into the question has divided opinions about whether games exert some influence on human behaviors and attitudes in real life. One influential school of thought, which mainly focused on violent video games and their aggression effects, proposed that players’ in-game behaviors are associated with their psychological traits in real-life, and playing videogames can produce psychological changes (Anderson and Bushman, 2001; Bushman and Anderson, 2002; Wiegman and van Schie, 1998).

The investigation into the aggression effects of violent games mainly relied on the General Aggression Model (GAM), proposed by Allen et al. (2018). In short, this framework argues that repetitive exposure to violent content increases the chance of accessing violent concepts, which induce the players to form aggressive thoughts and emotions, but the magnitude of this effect is dependent on the players’ traits, such as their personality (Allen et al. 2018). Even though an analysis showed that the aggression effect of violent games was found in numerous experimental, cross-sectional, and longitudinal studies (Anderson et al. 2010), its result is subjected to significant publication bias presented in empirical research, and the aggression effect decreased substantially upon the removal of this bias (Hilgard et al. 2017). The GAM framework was criticized for different reasons; one of the most notable critiques is that it underestimates the human ability to distinguish between reality and the virtual world (Ferguson and Dyck, 2012). The capacity to separate reality and the virtual world can cause the players to exhibit in-game behaviors and attitudes that do not align with their attitudes and behaviors in the real world. For example, Hartmann (2017) found that players who believed killing humans to be immoral in real life were fine with killing human characters in-game. In addition, numerous studies also found no association between playing violent games and aggression among children (Przybylski and Weinstein, 2019; Wiegman and van Schie, 1998). It was argued that the players’ psychological traits in real life and factors other than game content that affect their in-game behaviors, not vice versa (Adachi and Willoughby, 2011; Breuer et al. 2015; Cherryholmes, 1966; Garvey and Seiler, 1966).

Despite this, digital game-based learning research, including environmental education, argued that video games are a promising tool to facilitate attitudinal and behavioral changes for reasons. First, games offer better flexibility to incorporate stimulation in learning than traditional methods (Pentz et al. 2019) and are regarded as a potential substitute for communicating environmental knowledge (Janakiraman et al. 2018). Ahn et al. (2014) found that experience in immersive virtual environments produced better results on pro-environmental behaviors than print and videos. Some games allow players to experience as if they are in the physical world (Ahn et al. 2014), and virtual nature experience may develop emotions and increase nature connectedness, especially when access to physical nature is not always available (Yeo et al. 2020). In a virtual world that illustrates real-world problems, players also learn about environmental issues (e.g., climate change) and the solutions to address or mitigate them (Meya and Eisenack, 2018; Sterman et al. 2015). Exposure to virtual nature could also engage players in pro-environmental behaviors, especially when players are exposed to a situation where nature is destroyed (Klein and Hilbig, 2018). Second, by setting rules and goals, games help develop and sustain attitudinal change by motivating players to execute an action that targets desirable attitudes (Reigeluth, 2013). For example, using the daily energy consumption of an office to determine in-game rewards for “Energy Chickens,” Orland et al. (2014) showed that the office’s energy consumption was reduced by 13%. Participants also claimed to be more energy conscious even outside the office. In another study, which used the energy consumption of a virtual house, game-playing produced a similar decrease in real-life energy consumption (Gangolells et al. 2020). Further studies reported that games could improve players’ perceptions and behaviors towards environmental issues: recycling (Centieiro et al. 2011), energy waste (Ro et al. 2017), paper waste (Ahn et al. 2014). However, in-game behavior could induce pro-environmental attitudes and behavior with the outcome depending largely on the game mechanics. Therefore, an investigation of the motivation underlying pro-environmental behaviors is a necessary step to develop suitable strategies during the game designing process (Houdt et al. 2020).

### Economic rationales of Animal Crossing: New Horizon

The mechanics of ACNH offers a good platform to explore the correlation between in-game behaviors of ACNH players and their perception of resource scarcity. The virtual island in ACNH operates based on the economic principle of production and trade. The resources on the island, such as its flora and fauna, fossils, minerals (gold and iron), serve as the players’ source of income. These raw materials can be sold, or players could use them for crafting. However, these resources are limited. Even though most resources will reproduce regularly, the process takes some time, so players could only extract a certain amount at a time. When players have limited time, they need to optimize how they use this amount of time to collect items and use the extracted resources.

Furthermore, players also face the risk of losing the source of resources if it is over-exploited. For example, trees will not regrow if they are chopped down (for space or accidentally hit three times when collecting woods). Players could grow another tree by burying fruit, but that would take even more time for the tree to be ready for harvest. Meanwhile, unlike trees, flowers grow without a limit and players’ intervention. The game also encourages players to plant trees and flowers, rewarded with Nook Miles (for exchanging items, bells, etc.) and other benefits (Mateer and O’Roark, 2020).

Concerning planting activities in ACNH, we also investigate the purpose of growing and exploiting tree products and flowers. Table 1 summarizes activities using trees and flowers in the game.

**Table 1:** Benefits and purposes of planting trees and flowers.

	Tree			Flower
Product	Fruit	Wood	Tree	Flower
How to collect and the amount of time to grow back	Pick from trees. It takes 3 days to grow native fruit and up to 5–6 days to grow non-native ones	Use an ax to hit the tree; up to three logs could drop out of each tree. No definite time to be replenished	Burying fruit takes three days to grow and another day to have fruits	Pick up. There is no limit to the number of flowers growing per day. To grow hybrid types, flowers of different breeds must be planted in checkboard patterns to increase the chance.
Activities and benefits	Sell for money (100 bells for native fruit, 500 bells for non-native fruits)	Sell for money	N/A	Sell for money
	Material for crafting	Material for crafting	N/A	Material for crafting
	Eat to have energy used for relocating trees	N/A	N/A	Crossbreeding
	Use to grow new trees	N/A	N/A	Use to grow new flowers
	N/A	N/A	Chop down for space	Pick up for space
	N/A	N/A	N/A	Show off (wearing as accessories, decoration, etc.)
	N/A	N/A	Reserve for bugs, which are sold for money or donated (the stumps left after trees are cut down is home to some bugs)	Reserve for bugs, which are sold for money or donated
	Send gifts	N/A	N/A	Send gifts
	Esthetic purpose	N/A	Esthetic purpose	Esthetic purpose

Since in-game resources appear to be limited when there are many needs and activities, players may have to enhance the resources to satisfy various needs. Meanwhile, players may understand how acute the limit of resources is after investing time and efforts to replenish or increase these resources. For these reasons, the paper asks the following question: Is the perception of limited resources correlated with creating more resources?

## Methods

### Material and collecting procedure

This paper analyses a sample of 640 ACNH game players, collected through a convenient sampling with a global online survey from 15 to 30 May 2020. The survey was disseminated to multiple communities of ACNH players on social media, including Facebook, Reddit, and Discord (see Table S1). It aims to collect information about players’ demographic traits, their opinions in various statements regarding nature and human–nature relationships, and their behaviors when playing ACNH.

This section explains the data collection process in further detail. The collection process is divided into three steps to ensure that it complies with the community rules and that the participants’ rights are respected. In the first step, we conducted a pilot test with 15 ANCH players in Japan, Singapore, the USA, and Vietnam for their feedback on the contents of the survey and its appropriateness. Then, the authors contacted the admins and moderators for their permission and confirmation of standards and rules compliance before publicizing the survey in any online community. The survey was posted online with the admins/moderators’ confirmation for complying with the communities’ rules and standards. In the post, we also stated our purposes and briefly explained the survey contents so that participants could understand the situation. Moreover, we provided $5 Amazon gift cards to the first 100 respondents and 2$ Amazon gift cards to the subsequent 200 respondents as thank-you presents. Before answering the questions, all participants were required to read and fill in a consent form regarding confidentiality, the handling of research data, the disclosure of research outcomes.

In total, 640 geographically diverse ACNH players participated in the survey. 55% of respondents were from Canada and the USA. 28.13%, 14.38%, and 2.50% were from Asia, Europe, and other continents (i.e., South America, Oceania). The average age was 26.1%, and the portion of female respondents was 64.38%.

### Bayesian multilevel model

This paper employs the Bayesian multilevel model to assess the correlation between players’ environmental perception and their in-game interaction with flora and fauna of the virtual islands. There are both philosophical and practical debates regarding the Bayesian versus frequentist approach. Readers can explore both sides’ arguments from these suggested works (Kruschke, 2021; Kruschke and Liddell, 2018; McElreath, 2020; Pek and Van Zandt, 2019; van de Schoot and Depaoli, 2014; Vuong, Ho, & La, 2019). In the context of this paper, Bayesian statistics was chosen for its handling of small sample size and the incorporation of uncertainty in the estimation of the posterior distribution (Kruschke, 2021; Pek and Van Zandt, 2019; van de Schoot and Depaoli, 2014; Vuong et al. 2020). Furthermore, the Bayesian multilevel model was used for data analysis because of its utilization of partial pooling (McElreath, 2020). According to McElreath (2020), the partial pooling technique helps to explore the variation better, which is especially helpful with a geographically diverse dataset as used in this article. Moreover, as some of the variables in the analysis are construct variables, the partial pooling technique and Bayesian multilevel model will help analyze the averaging variables while still maintaining the variation in the original values. Finally, as the dataset is multinational, with participants from around the world with diverse ethnicity, the multilevel model can help adjust the estimations for both repeat sampling and imbalance in sampling.

The Bayesian multilevel models were performed with the *rethinking* package (version 2.13) in *R* statistical software (version 4.0.2).

### Variable description and data pre-processing

Table 2 provides a detailed description of the variables being used in this paper. Examples of R code to construct the models are available in the Supplementary Files.

**Table 2:** Outcome and predictor variables.

Variable type	Variable	Scale	Description
Outcome variable	Perception	Strongly disagree = 1; Disagree = 2; Not sure = 3; Agree = 4; Strongly agree = 5	The environmental perception of limited resources. The average of two items (C1 and C11) on the revised New Ecological Paradigm Scale.
Predictor variable	Plant Tree	Never = 1 to often = 4	The frequency that a game player plants a new tree
Predictor variable	Plant Flower	Never = 1 to often = 4	The frequency that a game player plants a new flower
Predictor variable	Develop Resources		The average frequency that a game player plants a new flower/tree
Predictor variable	Exploit Tree		The average frequency that a game player exploits tree for various purposes (woods, profit, etc.)
Predictor variable	Exploit Flowers		The average frequency that a game player exploits tree for various purposes (showing off, profit, etc.)
Predictor variable	Ethnicity	Asian = 1,	Game players’ ethnicities.
		Black or African American = 2,	
		Hispanic or Latino = 3,	
		Caucasian = 4,	
		Native American or American Indian = 5,	
		Pacific Islander = 6,	
		Other = 7	

For measuring the level of environmental perception among game players regarding the limited resources on Earth, we employed the revised New Ecological Paradigm Scale (NEPS) by Dunlap et al. (2000). The NEPS consists of 15 items that address five different facets of environmental worldview: the reality of limits to growth, anti-anthropocentrism, nature’s balance, denial of exceptionalism, and the possibility of an eco-crisis. This paper focuses on the first dimension, the reality of limits to growth because all of its questions are closely related to the matter of concern. However, there is no unanimous consensus about which of the 15 items belong to this dimension even though it was originally designed to consist of three items (coded as C1, C6, and C11 in Vuong et al. (2021) and Dunlap et al. (2000)). The internal consistency of the three original items is relatively low, as Cronbach’s alpha was only 0.271, which is potentially due to global samples with vastly diverse nationality and ethnicity (Vikan et al. 2007). The internal consistency increased substantially to the acceptable level of reliability after item C6 (Cronbach’s alpha = 0.548).

As such, we conducted an exploratory factor analysis to determine the factor structure of the 15 items and then used confirmatory factor analysis (CFA) to test the fit-to-data for the original model proposed by Dunlap et al. (2000) and the model obtained from the previous exploratory factor analysis. Based on the original work of Dunlap et al. (2000), in both models, all items were forced into five factors. The EFA results showed the factor concerning the reality of limits to growth consists of three items (C1, C11, and C13), among which item C13, “The balance of nature is very delicate and easily upset,” was supposed to belong to other factor called the balance of nature (see Supplementary Table S2). The items of this factor have standardized loading ranged from 0.46 (for C13) to 0.62 (for C11), and a Cronbach alpha of 0.587. The CFA results of the original model, where the limits-to-growth factor consists of C1, C6, and C11, showed poorer data fit than that of the model retrieved from EFA, where the limits-to-growth factor consists of C1, C11, and C13. This was based on the comparison of the ratio of chi-square to a degree of freedom, the root mean square error of approximation (RMSEA), the standardized root mean square residual (SRMR), and the comparative fit index (CFI) between these model (see Supplementary Tables S3 and S4). In the original model, the C6 item was not significantly correlated with the factor, while the other two items were. However, item C13 did not have a solid theoretical foundation. As the results of these analyses, as well as the theoretical background of the revised New Ecological Paradigm Scale, raised much doubt about the validity of C6 and C13, we selected two items, C1, and C11, to measure the environmental perception of limited resources. The outcome variable, perception of limited resources, used in our paper was the average score of C1 and C11.

To measure various kinds of interaction between the game players and in-game resources, game players were asked to rate their frequency of engaging in various activities (e.g., cutting down the tree, growing new trees, creating new types of flowers) on a scale from 1 (never) to 4 (often). These activities are grouped into two major themes: exploiting and developing resources. Therefore, they were averaged to create new variables such as “Exploit Tree,” “Exploit Flower,” and “Develop Resources.” Below is the formula for constructing the new variables:

$${\rm {Develop}}\,{\rm {Resources}} = \displaystyle\frac{{{\rm {Planting}}\,{\rm {Tree}}\,{\rm {and}}\,{\rm {Flower}} - \mu _{{\rm {Planting}}\,{\rm {Tree}}\,{\rm {and}}\,{\rm {Flower}}}}}{{\sigma _{{\rm {Planting}}\,{\rm {Tree}}\,{\rm {and}}\,{\rm {Flower}}}}}$$

$$\begin{array}{l}{\rm {Exploit}}\,{\rm {Tree}} = \displaystyle\frac{{{\rm {Take}}\,{\rm {woods}} \,+\, {\rm {Cut}}\,{\rm {down}}\,{\rm {Tree}} \,+\, {\rm {Sell}}\,{\rm {for}}\,{\rm {profit}} \,+\, {\rm {Reserve}}\,{\rm {for}}\,{\rm {Bug}}}}{4}\\ \qquad\qquad\qquad = \,\displaystyle\frac{{{\rm {Exploit}}\,{\rm {Tree}} \,-\, \mu _{\rm {{Exploit}}\,{\rm {Tree}}}}}{{\sigma _{{\rm {Exploit}}\,{\rm {Tree}}}}}\end{array}$$

$$\begin{array}{l}{\rm {Exploit}}\,{\rm {Flower}} = \displaystyle\frac{{{\rm {Crossbreed}} \,+\, {\rm {Showing}}\,{\rm {off}} \,+\, {\rm {Sell}}\,{\rm {for}}\,{\rm {profit}} \,+\, {\rm {Send}}\,{\rm {as}}\,{\rm {a}}\,{\rm {gift}}}}{4}\\ \qquad\qquad\qquad\quad = \,\displaystyle\frac{{{{{\mathrm{Exploit}}}}\,{{{\mathrm{Flower}}}} \,-\, \mu _{{\rm {Exploit}}\,{\rm {Flower}}}}}{{\sigma _{{\rm {Exploit}}\,{\rm {Flower}}}}}\end{array}$$

### Model construction and comparison

This paper followed a three-step Bayesian analysis procedure: model construction, model fitting, and model interpretation and improvement. As culture plays a critical role in shaping individuals’ perception of the external world (Atran and Medin, 2008), the multilevel model was applied as it allows us to evaluate the effects of ethnics to which the respondents belong their environmental perception. We also ran a series of similar models, but with the effect of the respondent’s region (see Supplementary, section Results for models with multilevel by Region).

For model selection, the Widely Applicable Information Criterion (WAIC) was applied to compare models’ ability to predict new data (McElreath, 2020). Trace plots were then used to diagnose the model’s posterior coefficients with the best predicting performance. Table 3 lists the mathematical formula of the five examined models:

**Table 3:** Mathematical formula of examined models.

Model name	Formula
Plant Tree	${\rm {Perception}}\sim {\rm{Plant}}\,{\rm{Tree}}$
	$${\rm {Perception}}_i\sim {\rm {Normal}}\left( {{\rm {Plant}}\,{\rm {Tree}}_i,p_i} \right)$$
	$${\rm {logit}}(p_i)\sim \alpha _{{\rm {Ethnics}}[i]}$$
	$$\alpha _{{\rm {Ethnics}}\left[ i \right]}\sim {\rm {Normal}}(0,1.5)$$
Plant Flower	${\rm {Perception}}\sim {\rm {Plant}}\,{\rm {Flower}}$
	$${\rm {Perception}}_i\sim {\rm {Normal}}\left( {{\rm {Plant}}\,{\rm {Flower}}_i,p_i} \right)$$
	$${\rm {logit}}(p_i)\sim \alpha _{{\rm {Ethnics}}[i]}$$
	$$\alpha _{{\rm {Ethnics}}\left[ i \right]}\sim {\rm {Normal}}(0,1.5)$$
Develop Resources	${\rm {Perception}}\sim {\rm {Develop}}\,{\rm {Resources}}$
	$${\rm {Perception}}_i\sim {\rm {Normal}}\left( {{\rm {Develop}}\,{\rm {Resources}}_i,p_i} \right)$$
	$${\rm {logit}}(p_i)\sim \alpha _{{\rm {Ethnics}}[i]}$$
	$$\alpha _{{\rm {Ethnics}}\left[ i \right]}\sim {\rm {Normal}}(0,1.5)$$
Exploit Tree	${\rm {Perception}}\sim {\rm {Exploit}}\,{\rm {Tree}}$
	$${\rm {Perception}}_i\sim {\rm {Normal}}\left( {{\rm {Exploit}}\,{\rm {Tree}}_i,p_i} \right)$$
	$${\rm {logit}}(p_i)\sim \alpha _{{\rm {Ethnics}}[i]}$$
	$$\alpha _{{\rm {Ethnics}}\left[ i \right]}\sim {\rm {Normal}}(0,1.5)$$
Exploit Flower	${\rm {Perception}}\sim {\rm {Exploit}}\,{\rm {Flower}}$
	$${\rm {Perception}}_i\sim {\rm {Normal}}\left( {\rm {{Exploit}}\,{\rm {Flower}}_i,p_i} \right)$$
	$${\rm {logit}}(p_i)\sim \alpha _{{\rm {Ethnics}}[i]}$$
	$$\alpha _{{\rm {Ethnics}}\left[ i \right]}\sim {\rm {Normal}}(0,1.5)$$

In these models, the prior of $\alpha _{{\rm {Ethnics}}\left[ i \right]}$ is assumed to be a regulating one, i.e., normal(0, 1.5) (McElreath, 2020).

## Results

### Technical validation

The MCMC diagnosis criteria are reported in Table 4. The MCMC simulations for all models contain 2000 samples from 4 chains.

**Table 4:** MCMC diagnostic criteria for the models.

	Plant Tree		Plant Flower		Develop Resource		Exploit Tree		Exploit Flower	
	n_eff	Rhat4	n_eff	Rhat4	n_eff	Rhat4	n_eff	Rhat4	n_eff	Rhat4
Asian	1829	1	1867	1	1081	1	1401	1	1113	1
Black or African American	2102	1	2765	1	781	1	1507	1	1164	1
Hispanic or Latin	2312	1	2171	1	1069	1	869	1	790	1
Caucasian	1849	1	2024	1	1185	1	1588	1	1296	1
Native American or American Indian	1789	1	1636	1	1189	1	1384	1	1282	1
Pacific Islander	1640	1	2203	1	1269	1	1320	1	1470	1
Other	1740	1	2627	1	1256	1	1242	1	1602	1

**Fig. 1:**
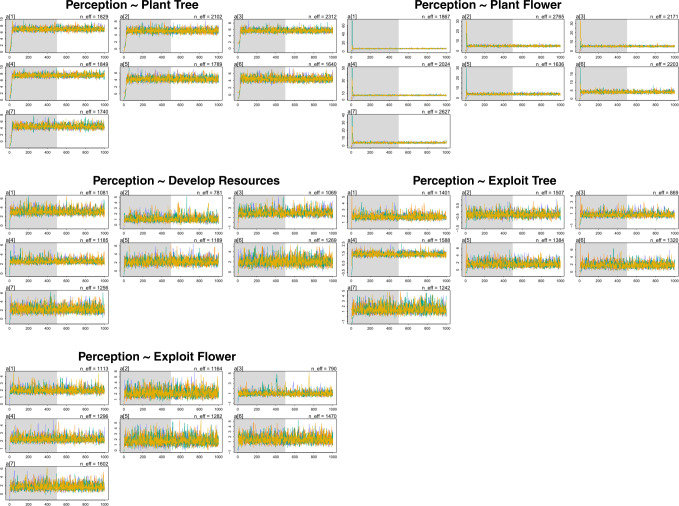
The trace plots for the models. Note: Asian = 1, Black or African American = 2, Hispanic or Latino = 3, Caucasian = 4, Native American or American Indian = 5, Pacific Islander = 6, Other = 7.

Most of them show that the effective sample size is >1000 (n_eff > 1000), and the Rhat4 = 1. This indicates that the posterior coefficients are well converged. For further technical validation of each model, we used different plots to visually diagnose the MCMC simulation. The models’ pairs plots (see Supplementary, Fig. S1) also provide more information on the posterior density of each parameter in the models and correlations between each parameter. Figure 1 suggests that the Markov chains in these plots are stationary and well-mixing around the same values, indicating a good convergence (McElreath, 2020; Vuong et al. 2020).

**Fig. 2:**
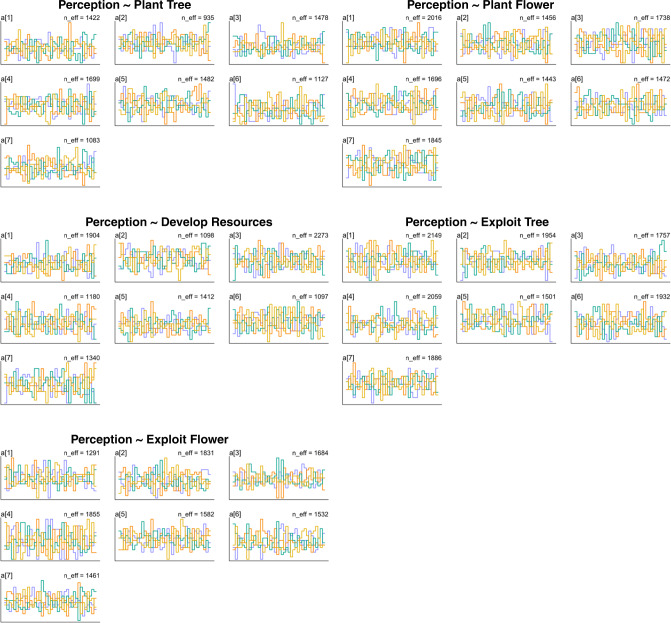
The trace rank plots for the models. Note: Asian = 1, Black or African American = 2, Hispanic or Latino = 3, Caucasian = 4, Native American or American Indian = 5, Pacific Islander = 6, Other = 7.

**Fig. 3:**
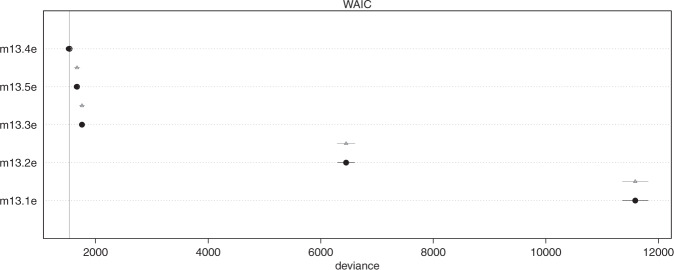
Model comparison.

Furthermore, the trace rank plots (as shown in Fig. 2) also provide a clear presentation of the MCMC chains. For each model, the histograms of rank frequency do not stray away from its ranges (Kruschke, 2021; McElreath, 2020), confirming the healthy chains that the trace plots in Fig. 3 indicate.

As all signals indicate the reliability of the models, widely applicable information criteria (WAIC) was used to compare between five models. The results are shown in Table 5, with visualization in Fig. 3. The model with Exploit Tree as the predictor has the lowest WAIC (1536.1) and the highest weight (1), which indicates this model should produce the best prediction, given the out-of-sample deviance (McElreath, 2020; Vehtari et al. 2017; Watanabe and Opper, 2010).

**Table 5:** Model comparison.

	WAIC	SE	dWAIC	dSE	pWAIC	weight
Exploit Tree	1536.1	50.85	0.0	NA	9.7	1
Exploit Flower	1669.9	46.54	133.8	52.04	6.1	0
Develop Resource	1757.9	39.74	221.8	48.88	3.6	0
Plant Flower	6450.9	151.61	4914.8	157.75	0.9	0
Plant Tree	11,588.7	227.07	10,052.6	229.43	0.9	0

### Model estimation

The paper used Bayesian multilevel models to account for the variation among ethnicity, given the predictor and outcome. Thus, Table 6 presents the posterior distributions, in terms of mean and standard deviation, for seven intercept parameters. The first three models have predictors as in-game developing actions (Plant Tree, Plant Flower, and Develop Resource), while the latter two indicate exploiting actions in ACNH (Exploit Tree and Exploit Flower).

Regarding the develop groups of models, the estimated posterior coefficients show that people with Asian or Caucasian ethnicities have the highest positive correlation between their in-game actions regarding development and their limit perception, with the mean ranging from 6.25 to 7.51 and the standard deviation ranging from 0.53 to 0.62 (Credible interval or CI is 95%). Meanwhile, other ethnic groups have lower positive correlations, ranging from 3.77 to 5.28 in the mean (s.d. ranges from 0.57 to 0.75, 95% CI) (Table 6).

**Table 6:** The posterior distributions of the models.

	Plant Tree		Plant Flower		Develop Resource		Exploit Tree		Exploit Flower	
	Mean	s.d.	Mean	s.d.	Mean	s.d.	Mean	s.d.	Mean	s.d.
Asian	7.01	0.57	6.25	0.60	3.10	0.65	1.91	0.40	2.01	0.42
Black or African American	5.28	0.62	4.90	0.67	1.13	0.58	−0.27	0.25	2.17	0.75
Hispanic or Latin	5.68	0.57	5.15	0.62	2.02	0.66	0.85	0.41	1.03	0.48
Caucasian	7.51	0.53	6.91	0.57	2.61	0.50	1.45	0.20	2.26	0.39
Native American or American Indian	4.30	0.71	3.92	0.75	2.26	0.84	1.67	0.83	1.83	0.81
Pacific Islander	4.45	0.69	4.05	0.70	2.13	0.84	1.88	0.80	1.54	0.79
Other	4.53	0.67	3.77	0.71	2.27	0.86	1.49	0.84	1.79	0.80

**Fig. 4:**
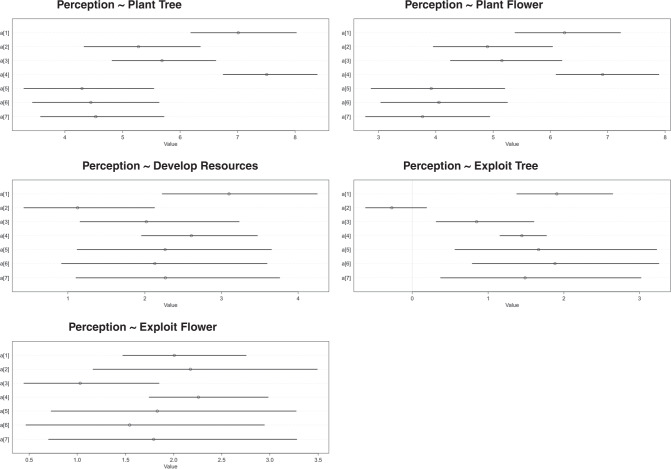
Visualization of posterior distributions of the models. Note: Asian = 1, Black or African American = 2, Hispanic or Latino = 3, Caucasian = 4, Native American or American Indian = 5, Pacific Islander = 6, Other = 7.

In the Exploit group of models, there are some interesting observations. First, according to Table 5, these models have lower WAIC compared to the Develop group of models, which indicates a higher predictive power. Second, for the model with Exploit Tree as the predictor, the action of exploiting tree in the Black and African American group has a negative correlation with the limit perception (mean = −0.27, s.d. = 0.25, with 95% credible intervals). The posterior distribution for the parameter of Caucasian ethnicity in this model also has one of the lowest standard deviations (0.20), with the mean of 1.45. The results of the model with the highest weight suggest a high level of confidence. The results are visualized in Fig. 4.

## Discussion

The article focuses on five in-game behaviors: planting trees and flowers, developing resources, and exploiting trees and flowers. The analysis suggests that people who plant trees and flowers or develop resources in a virtual environment potentially care about the Earth’s limited resources, with variations of different ethnicities. Similarly, the posterior distributions of Exploit Tree and Exploit Flower are also positively correlated with the perception of Earth’s limited resources, excluding the case of the Black or African American group in the Exploit Tree model.

Only two parameters in the Exploit Tree model have lower standard deviation: Black or African American (mean = −0.27, s.d. = 0.25) and Caucasian (mean = 1.45, s.d. = 0.20). The low standard deviations and the low WAIC of the Exploit Tree model suggest these results are technically more reliable. The negative correlation between Black or African Americans’ in-game action of exploiting trees and perception of limited resources suggests a potential relationship to be explored further. The correlations between in-game actions and perception of Earth’s limited resources indicate a viable option to utilize technology to raise awareness about nature and the environment. Results of previous studies have confirmed a link between being immersed in virtual nature and pro-environmental attitudes (Ahn et al. 2014; Hartmann and Apaolaza-Ibáñez, 2008); some found improvement in nature connectedness (Yeo et al. 2020). Many environmental movements focus on reforestation, such as Plant a Billion Trees (The Nature Conservancy, 2020), One Tree Planted (One Tree Planted, 2020), or Plant for the Planet (Plant for the Planet, 2020). Utilizing a virtual platform would help the movement reach out to more people, especially the younger population. Game companies such as Nintendo, Microsoft, or Sony can consider collaboration with these movements and other NGOs and NPOs (UNEP, 2019).

Aside from one difference, most of the posterior distributions in all models still fall into the positive range, with the standard deviations frequently ranging from 0.39 to 0.80. In reality, Pearse’s (1991) Pearse (1991) and Duguma and Hager’s (2009) results suggest that the perception of scarcity is associated with behaviors of enhancing or increasing resources. However, the behaviors in a virtual world may converge in the same direction regardless of the perception, potentially due to the existing rules and limitations of the games. In ACNH, players must bury fruit to grow a tree, and it takes 3 days to become a full-grown tree, while flowers can grow without any intervention. Furthermore, selling flowers do not produce as much profit as other products available on the island. 43 out of 53 types of flowers could be sold for 40–80 bells while selling fruit could obtain 100 bells per unit. Bugs and fish are also sold for a higher price. However, ACNH has almost no restriction or time limit on how to generate incomes, the players would have no pressure to maximize the profits in the shortest time. Hence, while there are differences, the players in ACNH eventually will do similar actions.

The indifference among actions, whether it is planting or exploiting trees and flowers, suggests that commercial game developers like ACNH might be able to provide more conscious environmental rules in the game world. These rules would create more in-game constraints that make gamers conscious of the values of doing related activities. For example, flowers should be granted more values and functions. Some disastrous environmental events should be added so that game players can become more aware of the virtually and realistically limited resources. By designing commercial games more appropriately, game developers might gain an opportunity to communicate about environmental issues (Woolbright, 2017) and initiate a reflection on reality (Lehner, 2017).

## Conclusion

Recently, video games have been argued to be a bridge between nature and humans, fostering nature connectedness (Bruni and Schultz, 2010; Schneider and Schaal, 2018). In a new normal after COVID-19, more people may stay in their homes and find comfort in a journey through a fictional but lively town on their virtual island. Our analysis suggests a viable opportunity to raise pro-environmental awareness through an entertainment product. However, the game developers, environment-related NGOs, and other stakeholders need to work together and incorporate pro-environmental elements.

The study is one of the earliest efforts, besides works like Mathevet et al. (2007), in evaluating the correlation between in-game behaviors and perception of limited resources. Thus, we fully acknowledge the limitations (Q.-H. Vuong, 2020).

Firstly, the sample size of 640 responses is representative but still small compared to the number of game players worldwide. Future studies with better resources can focus on expanding the sample size of the study. As we used a self-reported questionnaire to collect game players’ opinions, the provided answers can be unintentionally biased. Furthermore, the data were collected from several online communities, most of whom use English, so other communities are not covered in this survey. Hence, we suggest that future research expand the sample and take caution if the results are used for generalization.

Secondly, as we have pointed out in the literature review, both empirical results and theoretical foundations for the topic are limited. While this limitation allows us freedom to explore, it also hinders the ability to make meaningful interpretations of results. Indeed, the application of Bayesian multilevel models has helped with the exploration of variation in ethnicities. There is still more room for development and exploration. Thirdly, the study is conducted with only one game that we consider the most suitable. However, other games, such as farming games (Chang, 2012), or genres that focus heavily on resource management, could potentially offer the same experience and be included in future studies.

Finally, even though previous literature suggested that the perception–behavior relationship is interactive, we could only conclude that there may be a correlation between them due to data availability. Hence, we suggest future research to fill in this gap.

## Supplementary information

Supplementary for Virtual Tree, Real Impact: How Simulated Worlds Associate with the Perception of Limited Resources

## Data Availability

The dataset is available at Open Science Framework, URL: https://osf.io/p8u9c/; 10.17605/OSF.IO/P8U9C.
