# Supplementary material for: Virtual tree, real impact: how simulated worlds associate with the perception of limited resources
Source: Humanit Soc Sci Commun. 2022 Jun 24;9(1):213. doi: 10.1057/s41599-022-01225-1 (PMC9244435; doi:10.1057/s41599-022-01225-1)
Supplement: Supplementary file 1 — Supplementary for Virtual Tree, Real Impact: How Simulated Worlds Associate with the Perception of Limited Resources [file 41599_2022_1225_MOESM1_ESM.docx]

**Supplementary for**

**Virtual Tree, Real Impact: How Simulated Worlds Associate with the Perception of Limited Resources**

**Manh-Toan Ho ^1,2,*^, Thanh-Huyen T. Nguyen ^1^, Minh-Hoang Nguyen ^1^, Viet-Phuong La ^1^, Quan-Hoang Vuong ^1^**

^1^ Centre for Interdisciplinary Social Research, Phenikaa University, Yen Nghia Ward, Ha Dong District, Hanoi 100803, Vietnam

^2^ National Economics University, Hanoi, Vietnam

***** Corresponding Author: Manh-Toan Ho (toan.homanh@phenikaa-uni.edu.vn or toanhm.ephd5@st.neu.edu.vn)

**Table S1: Social media group**

| **Community** | **Platform** | **Starting date** |
| --- | --- | --- |
| Cộng đồng Nintendo Switch Việt Nam - SwitchVN Community | Facebook | 15th May 2020 |
| CLB địa chủ Animal Crossing New Horizons - Nintendo Switch Việt | Facebook | 15th May 2020 |
| Animal Crossing: New Horizons Over 25’s Club | Facebook | 19th May 2020 |
| Animal Crossing Community (UK) | Facebook | 24th May 2020 |
| Animal Crossing: New Horizons Philippines | Facebook | 25th May 2020 |
| Animal Crossing: New Horizons Myanmar 🇲🇲 | Facebook | 25th May 2020 |
| Animal Crossing: Arts and Codes (ACAC) | Facebook | 25th May 2020 |
| Animal Crossing: New Horizons | Discord | 24th May 2020 |
| r/Animal Crossing | Reddit | 19th May 2020 |

**Table S2: The results from exploratory factor analysis**

| > print(fa.none) Factor Analysis using method = pa Call: fa(r = newdata, nfactors = 5, rotate = "varimax", max.iter = 100,  fm = "pa") | | | | | | | | | | | | |
| --- | --- | --- | --- | --- | --- | --- | --- | --- | --- | --- | --- | --- |
| Standardized loadings (pattern matrix) based upon correlation matrix | | | | | | | | | | | | |
|  | PA1 | PA2 | PA3 | PA4 | | PA5 | | h2 | | u2 | | com |
| C1 | -0.03 | 0.23 | 0.57 | -0.05 | | 0.17 | | 0.40 | | 0.06 | | 1.5 |
| C2 | 0.60 | 0.04 | -0.05 | 0.00 | | 0.01 | | 0.37 | | 0.63 | | 1.0 |
| C3 | 0.00 | 0.43 | 0.37 | 0.18 | | 0.11 | | 0.37 | | 0.63 | | 2.5 |
| C4 | 0.50 | -0.08 | 0.07 | -0.03 | | 0.03 | | 0.27 | | 0.73 | | 1.1 |
| C5 | 0.24 | 0.61 | 0.28 | 0.04 | | 0.00 | | 0.52 | | 0.48 | | 1.8 |
| C6 | 0.32 | -0.38 | 0.09 | -0.05 | | 0.17 | | 0.28 | | 0.72 | | 2.6 |
| C7 | 0.15 | 0.43 | 0.21 | 0.63 | | 0.00 | | 0.64 | | 0.36 | | 2.2 |
| C8 | 0.78 | 0.07 | 0.06 | -0.02 | | -0.07 | | 0.63 | | 0.37 | | 1.0 |
| C9 | 0.10 | 0.58 | 0.18 | 0.25 | | 0.45 | | 0.45 | | 0.55 | | 1.7 |
| C10 | 0.71 | 0.19 | -0.02 | 0.15 | | 0.07 | | 0.56 | | 0.44 | | 1.3 |
| C11 | -0.03 | 0.12 | 0.62 | 0.03 | | -0.05 | | 0.40 | | 0.60 | | 1.1 |
| C12 | 0.69 | 0.21 | -0.04 | 0.20 | | -0.01 | | 0.56 | | 0.44 | | 1.4 |
| C13 | 0.03 | 0.14 | 0.46 | 0.21 | | -0.15 | | 0.30 | | 0.70 | | 1.9 |
| C14 | 0.63 | 0.06 | -0.09 | 0.04 | | -0.46 | | 0.62 | | 0.38 | | 1.9 |
| C15 | 0.11 | 0.65 | 0.36 | 0.05 | | -0.01 | | 0.57 | | 0.43 | | 1.6 |
|  | | | | | | | | | | | | |
|  | | | PA1 | | PA2 | | PA3 | | PA4 | | PA5 | |
| SS loadings | | | 2.81 | | 1.83 | | 1.37 | | 0.61 | | 0.32 | |
| Proportion Var | | | 0.19 | | 0.12 | | 0.09 | | 0.04 | | 0.02 | |
| Cumulative Var | | | 0.19 | | 0.31 | | 0.40 | | 0.44 | | 0.46 | |
| Proportion Explained | | | 0.41 | | 0.26 | | 0.20 | | 0.09 | | 0.05 | |
| Cumulative Proportion | | | 0.41 | | 0.67 | | 0.87 | | 0.95 | | 1.00 | |
|  | | | | | | | | | | | | |
| Test of the hypothesis that 5 factors are sufficient. | | | | | | | | | | | | |
|  | | | | | | | | | | | | |
| The degrees of freedom for the null model are 105 and the objective function was 4.24 with Chi Square of 2684.05 | | | | | | | | | | | | |
| The degrees of freedom for the model are 40 and the objective function was 0.1 | | | | | | | | | | | | |
|  | | | | | | | | | | | | |
| The root mean square of the residuals (RMSR) is 0.02 | | | | | | | | | | | | |
| The df corrected root mean square of the residuals is 0.03 | | | | | | | | | | | | |
|  | | | | | | | | | | | | |
| The harmonic number of observations is 640 with the empirical chi square 38.29 with prob < 0.55 | | | | | | | | | | | | |
| The total number of observations was 640 with Likelihood Chi Square = 62.52 with prob < 0.013 | | | | | | | | | | | | |
|  | | | | | | | | | | | | |
| Tucker Lewis Index of factoring reliability = 0.977 | | | | | | | | | | | | |
| RMSEA index = 0.03 and the 90 % confidence intervals are 0.014 0.043 | | | | | | | | | | | | |
| BIC = -195.94 | | | | | | | | | | | | |
| Fit based upon off diagonal values = 1 | | | | | | | | | | | | |
| Measures of factor score adequacy | | | | | | | | | | | | |
|  | | | PA1 | | PA2 | | PA3 | | PA4 | | PA5 | |
| Correlation of (regression) scores with factors | | | 0.92 | | 0.82 | | 0.78 | | 0.71 | | 0.62 | |
| Multiple R square of scores with factors | | | 0.84 | | 0.67 | | 0.61 | | 0.50 | | 0.39 | |
| Minimum correlation of possible factor scores 0.68 0.33 0.22 -0.01 -0.23 | | | 0.68 | | 0.33 | | 0.22 | | -0.01 | | -0.23 | |

**Table S3: The results from confirmatory factor analysis for the original model in Dunlap et al. (2000)**

| **Estimator** | | | | | | ML | | |
| --- | --- | --- | --- | --- | --- | --- | --- | --- |
| **Optimization method** | | | | | | NLMINB | | |
| **Number of model parameters** | | | | | | 40 | | |
|  | | | | | |  | | |
| **Number of observations** | | | | | | 640 | | |
|  | | | | | |  | | |
| **Model Test User Model:** | | | | | |  | | |
|  | | | | | |  | | |
| **Test statistic** | | | | | | 1129.624 | | |
| **Degrees of freedom** | | | | | | 80 | | |
| **P-value (Chi-square)** | | | | | | 0.000 | | |
|  | | | | | |  | | |
| **Model Test Baseline Model:** | | | | | |  | | |
|  | | | | | |  | | |
| **Test statistic** | | | | | | 2713.021 | | |
| **Degrees of freedom** | | | | | | 105 | | |
| **P-value** | | | | | | 0.000 | | |
|  | | | | | |  | | |
| **User Model versus Baseline Model:** | | | | | |  | | |
|  | | | | | |  | | |
| **Comparative Fit Index (CFI)** | | | | | | 0.598 | | |
| **Tucker-Lewis Index (TLI)** | | | | | | 0.472 | | |
|  | | | | | |  | | |
| **Loglikelihood and Information Criteria:** | | | | | |  | | |
|  | | | | | |  | | |
| **Loglikelihood user model (H0)** | | | | | | -13064.807 | | |
| **Loglikelihood unrestricted model (H1)** | | | | | | -12499.995 | | |
|  | | | | | |  | | |
| **Akaike (AIC)** | | | | | | 26209.614 | | |
| **Bayesian (BIC)** | | | | | | 26388.072 | | |
| **Sample-size adjusted Bayesian (BIC)** | | | | | | 26261.075 | | |
|  | | | | | |  | | |
| **Root Mean Square Error of Approximation:** | | | | | |  | | |
|  | | | | | |  | | |
| **RMSEA** | | | | | | 0.143 | | |
| **90 Percent confidence interval - lower** | | | | | | 0.136 | | |
| **90 Percent confidence interval - upper** | | | | | | 0.151 | | |
| **P-value RMSEA <= 0.05** | | | | | | 0.000 | | |
|  | | | | | |  | | |
| **Standardized Root Mean Square Residual:** | | | | | |  | | |
|  | | | | | |  | | |
| **SRMR** | | | | | | 0.138 | | |
|  | | | | | |  | | |
| **Parameter Estimates:** | | | | | |  | | |
|  | | | | | |  | | |
| **Standard errors** | | | | | | Standard | | |
| **Information** | | | | | | Expected | | |
| **Information saturated (h1) model** | | | | | | Structured | | |
|  | | | | | |  | | |
| **Latent Variables:** |  |  |  |  |  | |  |  |
|  |  | **Estimate** | **Std.Err** | **z-value** | **P(>\|z\|)** | | **Std.lv** | **Std.all** |
| **limit =~** |  |  |  |  |  | |  |  |
|  | **C1** | 1000 | NA | NA | NA | | 0.662 | 0.661 |
|  | **C6** | -0.033 | 0.086 | -0.381 | 0.703 | | -0.020 | -0.019 |
|  | **C11** | 0.986 | 0.149 | 6.597 | 0.000 | | 0.613 | 0.575 |
| **balance =~** |  |  |  |  |  | |  |  |
|  | **C3** | 1.000 | NA | NA | NA | | 0.193 | 0.222 |
|  | **C13** | 0.591 | 0.121 | 4.869 | 0.000 | | 0.114 | 0.117 |
|  | **C8** | 2.449 | 0.296 | 8.284 | 0.000 | | 0.473 | 0.363 |
| **ecocrisis =~** |  |  |  |  |  | |  |  |
|  | **C5** | 1.000 | NA | NA | NA | | 0.475 | 0.554 |
|  | **C10** | 1.742 | 0.142 | 12.236 | 0.000 | | 0.828 | 0.649 |
|  | **C15** | 0.865 | 0.086 | 10.029 | 0.000 | | 0.411 | 0.488 |
| **antiexempt =~** |  |  |  |  |  | |  |  |
|  | **C4** | 1.000 | NA | NA | NA | | 0.339 | 0.323 |
|  | **C9** | 0.993 | 0.137 | 7.262 | 0.000 | | 0.337 | 0.402 |
|  | **C14** | 1.693 | 0.219 | 7.726 | 0.000 | | 0.574 | 0.468 |
| **antianthro =~** |  |  |  |  |  | |  |  |
|  | **C2** | 1.000 | NA | NA | NA | | 0.602 | 0.560 |
|  | **C7** | 0.630 | 0.072 | 8.771 | 0.000 | | 0.379 | 0.430 |
|  | **C12** | 1.623 | 0.129 | 12.578 | 0.000 | | 0.977 | 0.747 |
|  |  |  |  |  |  | |  |  |
| **Covariances:** |  |  |  |  |  | |  |  |
|  |  | **Estimate** | **Std.Err** | **z-value** | **P(>\|z\|)** | | **Std.lv** | **Std.all** |
| **limit ~~** |  |  |  |  |  | |  |  |
|  | **balance** | 0.101 | 0.019 | 5.336 | 0.000 | | 0.843 | 0.843 |
|  | **ecocrisis** | 0.134 | 0.023 | 5.694 | 0.000 | | 0.452 | 0.452 |
|  | **antiexempt** | 0.047 | 0.019 | 2.447 | 0.014 | | 0.222 | 0.222 |
|  | **antianthro** | 0.020 | 0.024 | 0.024 | 0.833 | | 0.405 | 0.054 |
| **balance ~~** |  |  |  |  |  | |  |  |
|  | **ecocrisis** | 0.171 | 0.022 | 7.724 | 0.000 | | 1.868 | 1.868 |
|  | **antiexempt** | 0.131 | 0.021 | 6.315 | 0.000 | | 1.994 | 1.994 |
|  | **antianthro** | 0.191 | 0.026 | 7.436 | 0.000 | | 1.641 | 1.641 |
| **ecocrisis ~~** |  |  |  |  |  | |  |  |
|  | **antiexempt** | 0.191 | 0.026 | 7.259 | 0.000 | | 1.183 | 1.183 |
|  | **antianthro** | 0.258 | 0.029 | 8.822 | 0.000 | | 0.900 | 0.900 |
| **antiexempt ~~** |  |  |  |  |  | |  |  |
|  | **antianthro** | 0.233 | 0.032 | 7.172 | 0.000 | | 1.142 | 1.142 |
|  |  |  |  |  |  | |  |  |
| **Variances:** |  |  |  |  |  | |  |  |
|  |  | Estimate | Std.Err | z-value | P(>\|z\|) | | Std.lv | Std.all |
|  | **.C1** | 0.498 | 0.064 | 7.829 | 0.000 | | 0.498 | 0.563 |
|  | **.C6** | 1.121 | 0.063 | 17.884 | 0.000 | | 1.121 | 1.000 |
|  | **.C11** | 0.761 | 0.070 | 10.874 | 0.000 | | 0.761 | 0.669 |
|  | **.C3** | 0.718 | 0.042 | 17.123 | 0.000 | | 0.718 | 0.951 |
|  | **.C13** | 0.938 | 0.052 | 17.951 | 0.000 | | 0.938 | 0.986 |
|  | **.C8** | 1.472 | 0.113 | 12.998 | 0.000 | | 1.472 | 0.868 |
|  | **.C5** | 0.509 | 0.032 | 16.057 | 0.000 | | 0.509 | 0.693 |
|  | **.C10** | 0.943 | 0.066 | 14.278 | 0.000 | | 0.943 | 0.579 |
|  | **.C15** | 0.541 | 0.032 | 16.735 | 0.000 | | 0.541 | 0.762 |
|  | **.C4** | 0.986 | 0.056 | 17.516 | 0.000 | | 0.986 | 0.896 |
|  | **.C9** | 0.587 | 0.035 | 16.741 | 0.000 | | 0.587 | 0.838 |
|  | **.C14** | 1.175 | 0.075 | 15.603 | 0.000 | | 1.175 | 0.781 |
|  | **.C2** | 0.794 | 0.050 | 15.860 | 0.000 | | 0.794 | 0.687 |
|  | **.C7** | 0.632 | 0.037 | 16.926 | 0.000 | | 0.632 | 0.815 |
|  | **.C12** | 0.757 | 0.069 | 11.011 | 0.000 | | 0.757 | 0.442 |
|  | **limit** | 0.387 | 0.070 | 5.501 | 0.000 | | 1.000 | 1.000 |
|  | **balance** | 0.037 | 0.018 | 2.087 | 0.037 | | 1.000 | 1.000 |
|  | **ecocrisis** | 0.226 | 0.033 | 6.896 | 0.000 | | 1.000 | 1.000 |
|  | **antiexempt** | 0.115 | 0.030 | 3.875 | 0.000 | | 1.000 | 1.000 |
|  | **antianthro** | 0.362 | 0.052 | 6.918 | 0.000 | | 1.000 | 1.000 |

**Table S4: The results from confirmatory factor analysis for the model retrieved from EFA**

| **Estimator** | | | | | | | ML | |
| --- | --- | --- | --- | --- | --- | --- | --- | --- |
| **Optimization method** | | | | | | | NLMINB | |
| **Number of model parameters** | | | | | | | 36 | |
| **Number of observations** | | | | | | | 640 | |
|  | | | | | | | | |
| **Model Test User Model:** | | | | | | | | |
| **Test statistic** | | | | | | | 331.141 | |
| **Degrees of freedom** | | | | | | | 84 | |
| **P-value (Chi-square)** | | | | | | | 0.000 | |
|  | | | | | | | | |
| **Model Test Baseline Model:** | | | | | | | | |
| **Test statistic** | | | | | | | 2713.021 | |
| **Degrees of freedom** | | | | | | | 105 | |
| **P-value (Chi-square)** | | | | | | | 0.000 | |
|  | | | | | | | | |
| **User Model versus Baseline Model:** | | | | | | | | |
| **Comparative Fit Index (CFI)** | | | | | | | 0.905 | |
| **Tucker-Lewis Index (TLI)** | | | | | | | 0.882 | |
|  | | | | | | |  | |
| **Loglikelihood and Information Criteria:** | | | | | | | | |
| **Loglikelihood user model (H0)** | | | | | | | -12665.565 | |
| **Loglikelihood unrestricted model (H1)** | | | | | | | -12499.995 | |
|  | | | | | | | | |
| **Akaike (AIC)** | | | | | | | 25403.131 | |
| **Bayesian (BIC)** | | | | | | | 25563.744 | |
| **Sample-size adjusted Bayesian (BIC)** | | | | | | | 25449.446 | |
|  | | | | | | | | |
| **Root Mean Square Error of Approximation:** | | | | | | | | |
| **RMSEA** | | | | | | | 0.068 | |
| **90 Percent confidence interval - lower** | | | | | | | 0.060 | |
| **90 Percent confidence interval - upper** | | | | | | | 0.076 | |
| **P-value RMSEA <= 0.05** | | | | | | | 0.000 | |
|  | | | | | | | | |
| **Standardized Root Mean Square Residual:** | | | | | | | | |
| **SRMR** | | | | | | | 0.069 | |
|  | | | | | | | | |
| **Parameter Estimates:** | | | | | | | | |
|  | | | | | | | | |
| **Standard errors** | | | | | | | Standard | |
| **Information** | | | | | | | Expected | |
| **Information saturated (h1) model** | | | | | | | Structured | |
|  | | | | | | | | |
| **Latent Variables:** | | | | | | | | |
|  |  | **Estimate** | **Std.Err** | **z-value** | **P(>\|z\|)** | **Std.lv** | | **Std.all** |
| **limit =~** | | | | | | | | |
|  | **C1** | 1.000 |  |  |  | 0.554 | | 0.589 |
|  | **C11** | 1.159 | 0.123 | 9.416 | 0.000 | 0.643 | | 0.603 |
|  | **C13** | 0.878 | 0.103 | 8.556 | 0.001 | 0.487 | | 0.499 |
| **ecocrisis =~** | | | | | | | | |
|  | **C5** | 1.000 |  |  |  | 0.593 | | 0.691 |
|  | **C15** | 1.035 | 0.068 | 15.241 | 0.000 | 0.613 | | 0.728 |
|  | **C9** | 0.912 | 0.066 | 13.876 | 0.000 | 0.541 | | 0.646 |
|  | **C3** | 0.946 | 0.074 | 12.710 | 0.000 | 0.561 | | 0.645 |
|  | **C6** | -0.304 | 0.078 | -3.889 | 0.000 | -0.180 | | -0.170 |
| **antianthro =~** | | | | | | | | |
|  | **C7** | 1.000 |  |  |  | 0.881 | | 1.000 |
| **other =~** | | | | | | | | |
|  | **C8** | 1.000 |  |  |  | 0.971 | | 0.746 |
|  | **C10** | 0.989 | 0.057 | 17.404 | 0.000 | 0.961 | | 0.753 |
|  | **C12** | 0.987 | 0.058 | 16.998 | 0.000 | 0.959 | | 0.733 |
|  | **C3** | -0.124 | 0.038 | -3.294 | 0.001 | -0.120 | | -0.138 |
|  | **C14** | 0.801 | 0.054 | 14.798 | 0.000 | 0.778 | | 0.634 |
|  | **C2** | 0.666 | 0.047 | 14.033 | 0.000 | 0.646 | | 0.601 |
|  | **C4** | 0.503 | 0.046 | 10.868 | 0.000 | 0.489 | | 0.466 |
|  | | | | | | | | |
| **Covariances:** | | | | | | | | |
|  |  | **Estimate** | **Std.Err** | **z-value** | **P(>\|z\|)** | **Std.lv** | | **Std.all** |
| **limit =~** | | | | | | | | |
|  | **ecocrisis** | 0.226 | 0.027 | 8.416 | 0.000 | 0.687 | | 0.687 |
|  | **antianthro** | 0.187 | 0.028 | 6.563 | 0.000 | 0.383 | | 0.383 |
|  | **other** | 0.004 | 0.030 | 0.123 | 0.902 | 0.007 | | 0.007 |
| **ecocrisis ~~** | | | | | | | | |
|  | **antianthro** | 0.315 | 0.030 | 10.656 | 0.000 | 0.603 | | 0.603 |
|  | **other** | 0.199 | 0.032 | 6.264 | 0.000 | 0.346 | | 0.346 |
| **antianthro ~~** | | | | | | | | |
|  | **other** | 0.240 | 0.039 | 6.149 | 0.000 | 0.281 | | 0.281 |
|  | | | | | | | | |
| **Variances:** | | | | | | | | |
|  |  | **Estimate** | **Std.Err** | **z-value** | **P(>\|z\|)** | **Std.lv** | | **Std.all** |
|  | **.C1** | 0.577 | 0.044 | 13.138 | 0.000 | 0.577 | | 0.653 |
|  | **.C11** | 0.724 | 0.057 | 12.782 | 0.000 | 0.724 | | 0.637 |
|  | **.C13** | 0.714 | 0.048 | 14.991 | 0.000 | 0.714 | | 0.751 |
|  | **.C5** | 0.384 | 0.027 | 14.205 | 0.000 | 0.384 | | 0.522 |
|  | **.C15** | 0.334 | 0.025 | 13.345 | 0.000 | 0.334 | | 0.470 |
|  | **.C9** | 0.408 | 0.027 | 15.023 | 0.000 | 0.408 | | 0.582 |
|  | **.C3** | 0.473 | 0.031 | 15.138 | 0.000 | 0.473 | | 0.626 |
|  | **.C6** | 1.089 | 0.061 | 17.772 | 0.000 | 1.089 | | 0.971 |
|  | **.C7** | 0.000 |  |  |  | 0.000 | | 0.000 |
|  | **.C8** | 0.752 | 0.055 | 13.619 | 0.000 | 0.752 | | 0.444 |
|  | **.C10** | 0.706 | 0.053 | 13.432 | 0.000 | 0.706 | | 0.433 |
|  | **.C12** | 0.792 | 0.057 | 13.938 | 0.000 | 0.792 | | 0.463 |
|  | **.C14** | 0.899 | 0.058 | 15.622 | 0.000 | 0.899 | | 0.598 |
|  | **.C2** | 0.738 | 0.046 | 15.988 | 0.000 | 0.738 | | 0.639 |
|  | **.C4** | 0.862 | 0.051 | 16.964 | 0.000 | 0.862 | | 0.783 |
|  | **limit** | 0.307 | 0.048 | 6.416 | 0.000 | 1.000 | | 1.000 |
|  | **ecocrisis** | 0.351 | 0.039 | 9.081 | 0.000 | 1.000 | | 1.000 |
|  | **antianthro** | 0.776 | 0.043 | 17.889 | 0.000 | 1.000 | | 1.000 |
|  | **other** | 0.943 | 0.092 | 10.230 | 0.000 | 1.000 | | 1.000 |

**Figure S1: Pairs plots for Perception models in the article**

**
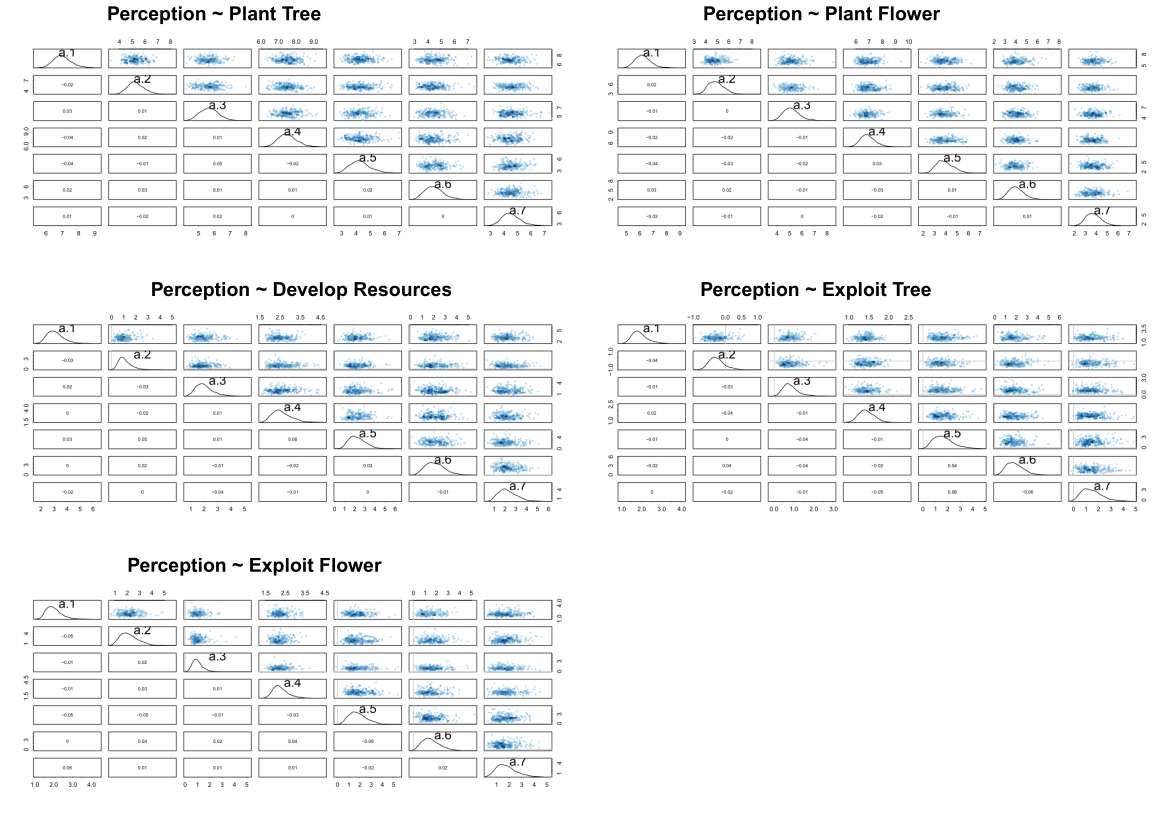
**

**Results for models with multilevel by Region**

In this section, we present the results for five models, which have similar outcome and predictor variables to the models in the paper. However, these models are multilevel by Region, rather than Ethnicity. There are four regions: Asia, EU, US/Canada, and Other. The posterior distributions of the Region models suggest similar results as the Ethnicity models. There are minor differences among region, but all of the posterior distributions are positive correlation.

**Table S5: MCMC diagnostic criteria for the Region models**

|  | **Plant Tree** | | **Plant Flower** | | **Develop Resource** | | **Exploit Tree** | | **Exploit Flower** | |
| --- | --- | --- | --- | --- | --- | --- | --- | --- | --- | --- |
|  | ***n_eff*** | ***Rhat4*** | ***n_eff*** | ***Rhat4*** | ***n_eff*** | ***Rhat4*** | ***n_eff*** | ***Rhat4*** | ***n_eff*** | ***Rhat4*** |
| **Asia** | 1887 | 1 | 1595 | 1 | 836 | 1 | 869 | 1 | 551 | 1 |
| **EU** | 1392 | 1 | 2020 | 1 | 861 | 1 | 1105 | 1 | 513 | 1 |
| **US/Canada** | 1645 | 1 | 1825 | 1 | 730 | 1 | 1083 | 1 | 507 | 1.01 |
| **Other** | 2115 | 1 | 1774 | 1 | 980 | 1 | 873 | 1 | 817 | 1 |

**Figure S2: Pairs plots for the Region models**

**
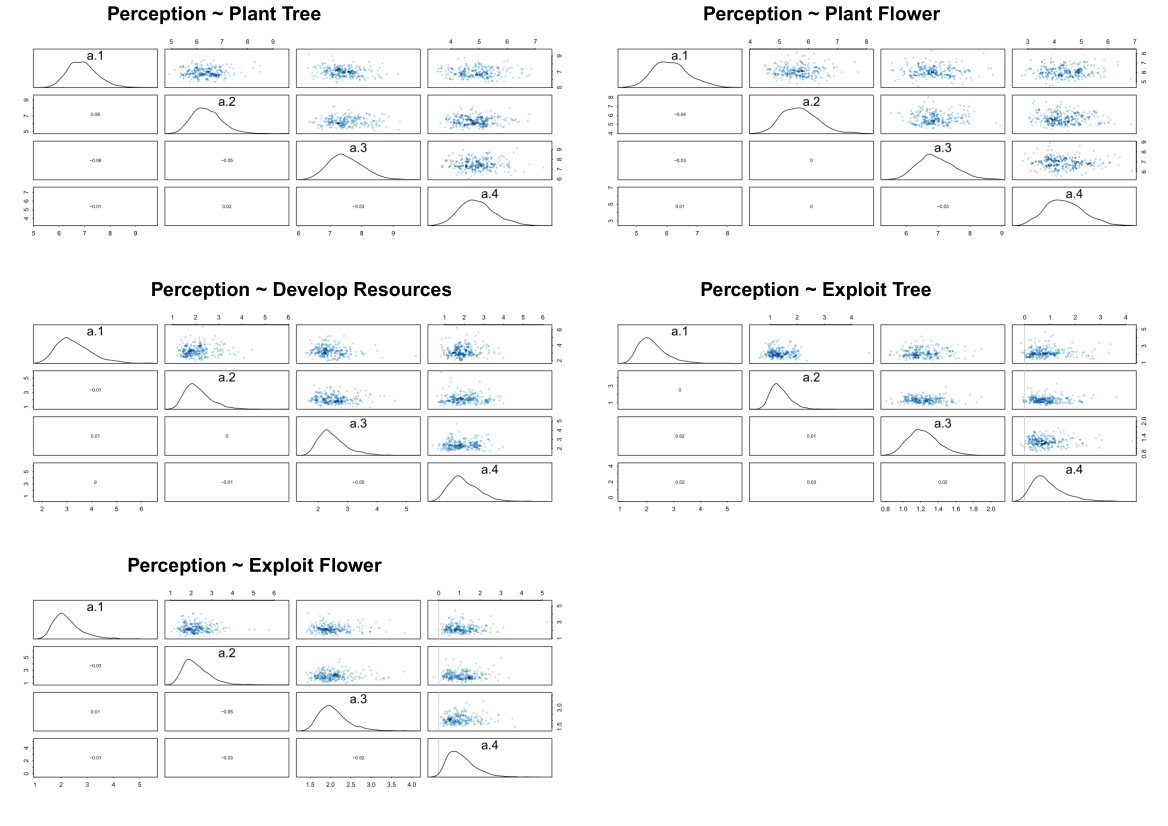
**

**Figure S3: The trace plots for the Region models**

**
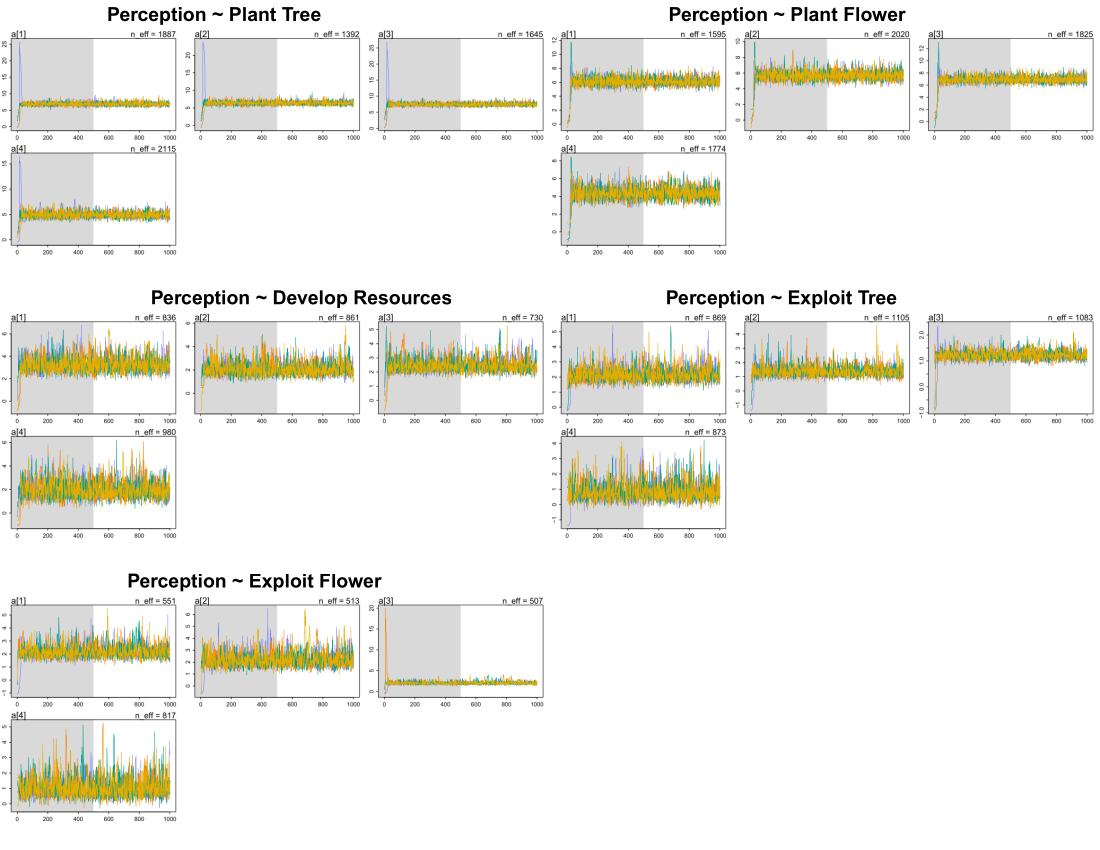
**

**Figure S4: The trace rank plots for the Region models**

**
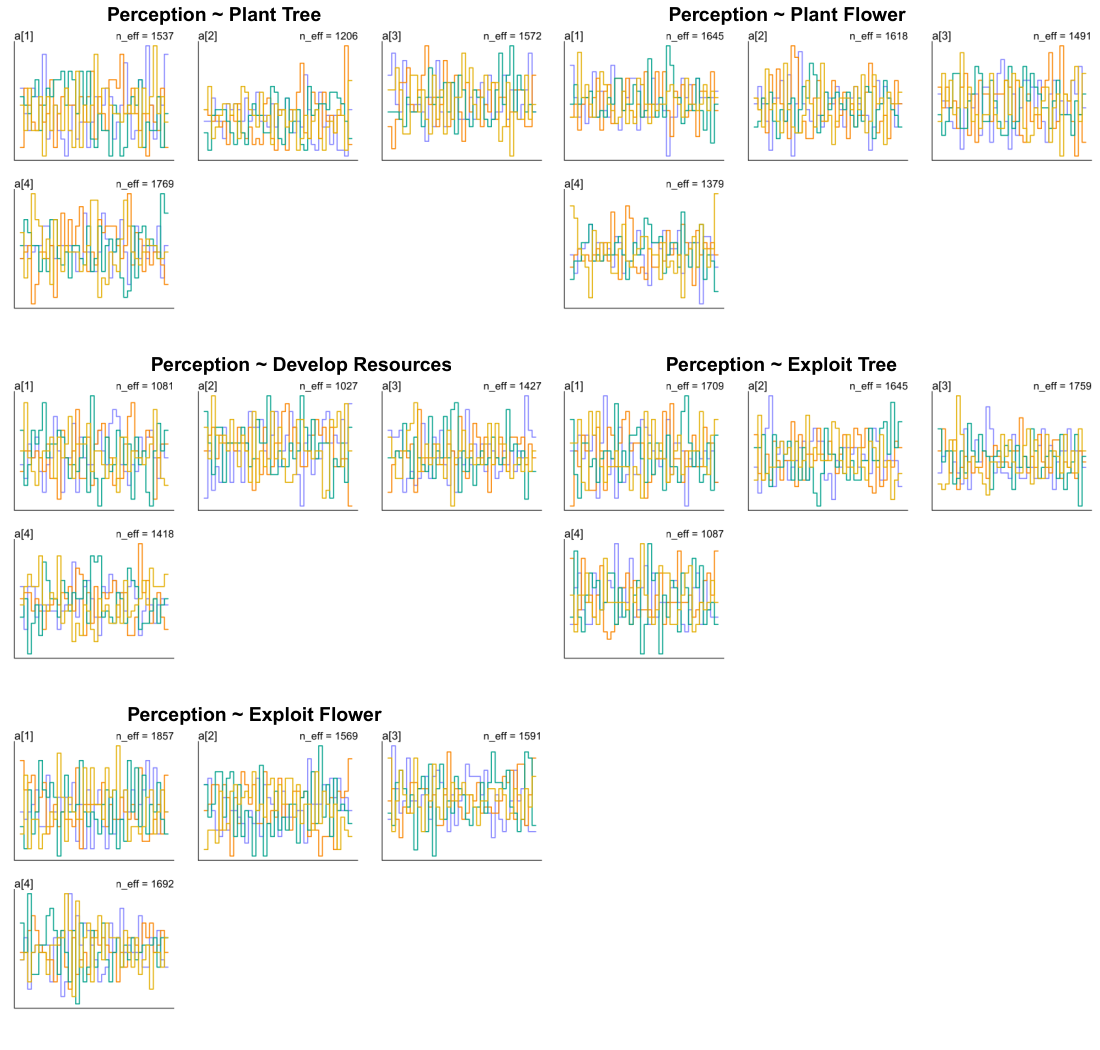
**

**Table S6: Model comparison for Region models**

|  | **WAIC** | **SE** | **dWAIC** | **dSE** | **pWAIC** | **weight** |
| --- | --- | --- | --- | --- | --- | --- |
| **Exploit Tree** | 1544.7 | 49.73 | 0.0 | NA | 5.5 | 1 |
| **Exploit Flower** | 1668.1 | 45.96 | 123.5 | 50.15 | 4.2 | 0 |
| **Develop Resource** | 1755.4 | 39.16 | 210.8 | 47.16 | 2.2 | 0 |
| **Plant Flower** | 6440.0 | 151.29 | 4895.4 | 155.78 | 0.2 | 0 |
| **Plant Tree** | 11576.4 | 226.82 | 10031.8 | 228.17 | 0.2 | 0 |

**Figure S5: Model comparison for Region models**

**
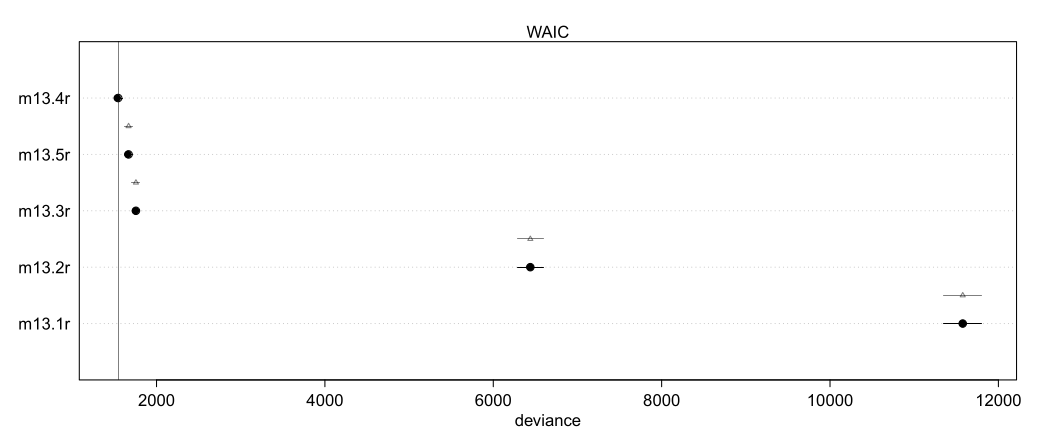
**

**Table S7: The posterior distributions of the Region models**

|  | **Plant Tree** | | **Plant Flower** | | **Develop Resource** | | **Exploit Tree** | | **Exploit Flower** | |
| --- | --- | --- | --- | --- | --- | --- | --- | --- | --- | --- |
|  | ***mean*** | ***sd*** | ***mean*** | ***sd*** | ***mean*** | ***sd*** | ***mean*** | ***sd*** | ***mean*** | ***sd*** |
| **Asia** | 6.93 | 0.59 | 6.15 | 0.60 | 3.30 | 0.70 | 2.21 | 0.53 | 2.25 | 0.55 |
| **EU** | 6.40 | 0.59 | 5.71 | 0.62 | 2.12 | 0.60 | 1.39 | 0.39 | 2.27 | 0.68 |
| **US/Canada** | 7.50 | 0.55 | 6.95 | 0.58 | 2.50 | 0.50 | 1.23 | 0.18 | 2.09 | 0.39 |
| **Other** | 4.94 | 0.63 | 4.38 | 0.71 | 2.01 | 0.77 | 0.92 | 0.64 | 1.11 | 0.69 |

**Figure S6: Visualization of posterior distributions of the Region models**

**
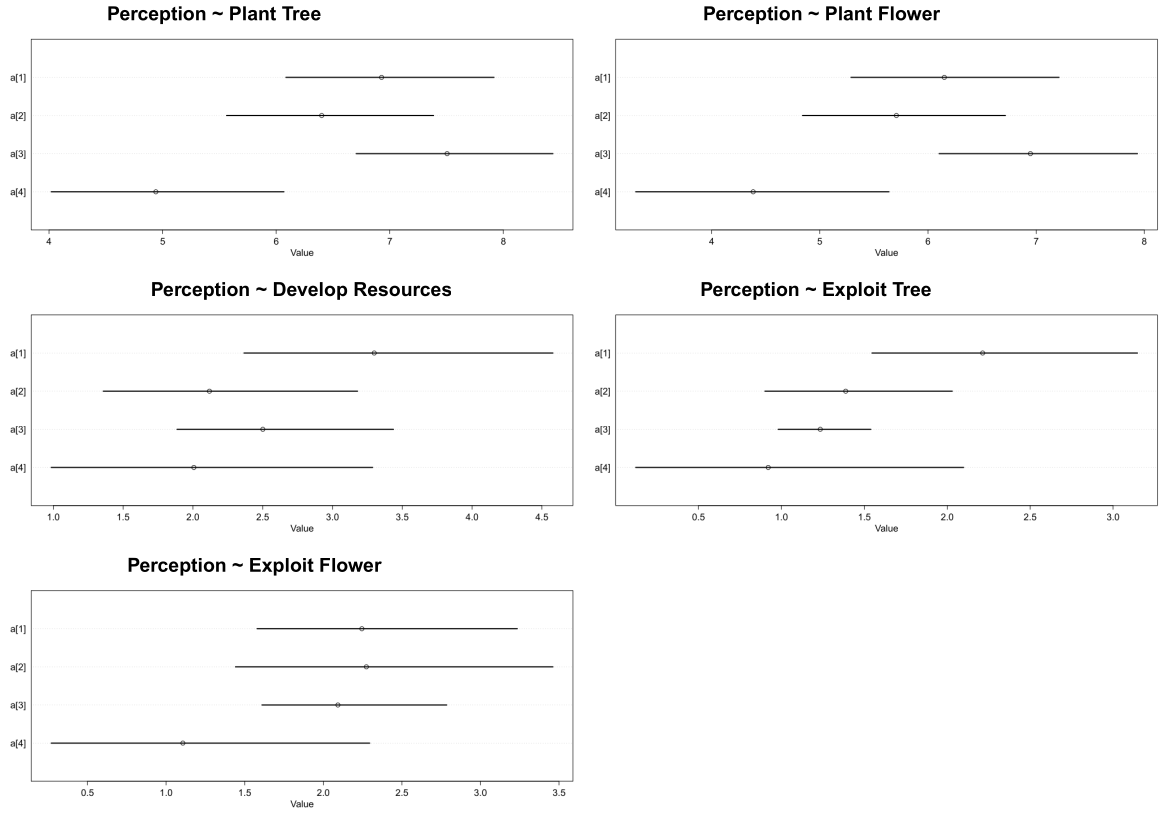
**

**Examples of R code**

| library(rstan)  library(devtools)  library(rethinking)  library(bayes)  library(dplyr)  library(readr)  options(mc.cores=parallel::detectCores())  d <- na.omit(d)  d <- read.csv("C:/Users/manht/Downloads/Data Run/Game 3/dat_640_cleaned.csv")  ##Variable manipulation###  ###1. Demography  Ethnic <-  case_when(  d$A6 %in% c("Asian") ~ 1,  d$A6 %in% c("Black or African American") ~ 2,  d$A6 %in% c("Hispanic or Latino") ~ 3,  d$A6 %in% c("White") ~4,  d$A6 %in% c("Native American or American Indian") ~5,  d$A6 %in% c("Pacific Islander") ~6,  d$A6 %in% c("Other") ~7,  )  Region <-  case_when(  d$Region %in% c("Asia") ~ 1,  d$Region %in% c("EU") ~ 2,  d$Region %in% c("US/Canada") ~ 3,  d$Region %in% c("Other") ~4,  )  Sex <-  case_when(  d$A2 %in% c("Male") ~ 1,  d$A2 %in% c("Female") ~ 0  )  ###2. Frequency of using in-game resources  exploit_tree <- (d$E16 + d$E17 + d$E18 + d$E19)/4  exploit_flower <- (d$E21 + d$E22 + d$E23 + d$E24)/4  exploit_tree_s <- (exploit_tree - mean(exploit_tree))/(sd(exploit_tree)) #Standardized exploiting tree variable  exploit_flower_s <- (exploit_flower - mean(exploit_flower))/(sd(exploit_flower)) #Standardized exploiting flower variable  exploit_resources_s <- (exploit_flower_s + exploit_tree_s)/2  ###3. Frequency of developing new resources  develop_resources_s <- (d$E3 - mean(d$E3))/(sd(d$E3)) #For both tree and flower  develop_tree <- d$E15  devlop_flower <- d$E20  ###4, Frequency of exploring new resources  finding_resources_s <- (d$E25 - mean(d$E25))/(sd(d$E25))  explore_new_species_s <- (d$E28 - mean(d$E28))/(sd(d$E28))  explore_resources <- (finding_resources_s + explore_new_species_s)/2  ###2. Outcome variable  item1_s <- (d$C1 - mean(d$C1))/(sd(d$C1))  item11_s <- (d$C11 - mean(d$C11))/(sd(d$C11))  response <- (item1_s + item11_s)/2  scarcity_perception_3items <- (d$C1 + d$C6 + d$C11)/3  scarcity_perception_C6_only <- d$C6  ### Model ###  ###Model for rethinking###  ###Ethnicity###  # detect the number of cores of your CPU  options(mc.cores = parallel::detectCores())  ##Limit ~ DevTree##  dat13.1e <-list(  O =as.integer(response),  T =as.integer(develop_tree),  E =as.integer(Ethnic))  m13.1e <- ulam(  alist(  O ~dnorm(T,p),  logit(p) <-a[E],  a[E] ~dnorm(0,1.5)  ), data=dat13.1e,chains=4,log_lik=TRUE)  labels <-paste("a[",1:7,"]:",levels(Ethnic),sep="")  plot( precis(m13.1e,depth=2,pars="a"),labels=labels)  precis(m13.1e,depth=2)  plot(precis(m13.1e,depth=2))  pairs(m13.1e, omit ="cutpoints")  traceplot(m13.1e)  trankplot(m13.1e)  # detect the number of cores of your CPU  options(mc.cores = parallel::detectCores())  ### Limit ~ DevFlower##  dat13.2e <-list(  O =as.integer(response),  F =as.integer(devlop_flower),  E =as.integer(Ethnic))  m13.2e <- ulam(  alist(  O ~dnorm(F,p),  logit(p) <-a[E],  a[E] ~dnorm(0,1.5)  ), data=dat13.2e,chains=4,log_lik=TRUE)  precis(m13.2e,depth=2)  plot(precis(m13.2e,depth=2))  pairs(m13.2e, omit ="cutpoints")  traceplot(m13.2e)  trankplot(m13.2e)  # detect the number of cores of your CPU  options(mc.cores = parallel::detectCores())  ### Limit ~ DevRes##  dat13.3e <-list(  O =as.integer(response),  DR =as.integer(develop_resources_s),  E =as.integer(Ethnic))  m13.3e <- ulam(  alist(  O ~dnorm(DR,p),  logit(p) <-a[E],  a[E] ~dnorm(0,1.5)  ), data=dat13.3e,chains=4,log_lik=TRUE)  precis(m13.3e,depth=2)  plot(precis(m13.3e,depth=2))  pairs(m13.3e, omit ="cutpoints")  traceplot(m13.3e)  trankplot(m13.3e)  # detect the number of cores of your CPU  options(mc.cores = parallel::detectCores())  ### Limit ~ ExploitTree##  dat13.4e <-list(  O =as.integer(response),  ET =as.integer(exploit_tree_s),  E =as.integer(Ethnic))  m13.4e <- ulam(  alist(  O ~dnorm(ET,p),  logit(p) <-a[E],  a[E] ~dnorm(0,1.5)  ), data=dat13.4e,chains=4,log_lik=TRUE)  precis(m13.4e,depth=2)  plot(precis(m13.4e,depth=2))  pairs(m13.4e, omit ="cutpoints")  traceplot(m13.4e)  trankplot(m13.4e)  # detect the number of cores of your CPU  options(mc.cores = parallel::detectCores())  ### Limit ~ ExploitFlower##  dat13.5e <-list(  O =as.integer(response),  EF =as.integer(exploit_flower_s),  E =as.integer(Ethnic))  m13.5e <- ulam(  alist(  O ~dnorm(EF,p),  logit(p) <-a[E],  a[E] ~dnorm(0,1.5)  ), data=dat13.5e,chains=4,log_lik=TRUE)  precis(m13.5e,depth=2)  plot(precis(m13.5e,depth=2))  pairs(m13.5e, omit ="cutpoints")  traceplot(m13.5e)  trankplot(m13.5e)  ##Region###  # detect the number of cores of your CPU  options(mc.cores = parallel::detectCores())  ##Limit ~ DevTree##  dat13.1r <-list(  O =as.integer(response),  T =as.integer(develop_tree),  R =as.integer(Region))  m13.1r <- ulam(  alist(  O ~dnorm(T,p),  logit(p) <-a[R],  a[R] ~dnorm(0,1.5)  ), data=dat13.1r,chains=4,log_lik=TRUE)  precis(m13.1r,depth=2)  Fig13.1r1 <- plot(precis(m13.1r,depth=2))  Fig13.1r2 <- pairs(m13.1r, omit ="cutpoints")  Fig13.1r3 <- traceplot(m13.1r)  Fig13.1r4 <- trankplot(m13.1r)  # detect the number of cores of your CPU  options(mc.cores = parallel::detectCores())  ### Limit ~ DevFlower##  dat13.2r <-list(  O =as.integer(response),  F =as.integer(devlop_flower),  R =as.integer(Region))  m13.2r <- ulam(  alist(  O ~dnorm(F,p),  logit(p) <-a[R],  a[R] ~dnorm(0,1.5)  ), data=dat13.2r,chains=4,log_lik=TRUE)  precis(m13.2r,depth=2)  Fig13.2r1 <- plot(precis(m13.2r,depth=2))  Fig13.2r1 <- pairs(m13.2r, omit ="cutpoints")  Fig13.2r1 <- traceplot(m13.2r)  Fig13.2r1 <- trankplot(m13.2r)  # detect the number of cores of your CPU  options(mc.cores = parallel::detectCores())  ### Limit ~ DevRes##  dat13.3r <-list(  O =as.integer(response),  DR =as.integer(develop_resources_s),  R =as.integer(Region))  m13.3r <- ulam(  alist(  O ~dnorm(DR,p),  logit(p) <-a[R],  a[R] ~dnorm(0,1.5)  ), data=dat13.3r,chains=4,log_lik=TRUE)  precis(m13.3r,depth=2)  plot(precis(m13.3r,depth=2))  pairs(m13.3r, omit ="cutpoints")  traceplot(m13.3r)  trankplot(m13.3r)  # detect the number of cores of your CPU  options(mc.cores = parallel::detectCores())  ### Limit ~ ExploitTree##  dat13.4r <-list(  O =as.integer(response),  ET =as.integer(exploit_tree_s),  R =as.integer(Region))  m13.4r <- ulam(  alist(  O ~dnorm(ET,p),  logit(p) <-a[R],  a[R] ~dnorm(0,1.5)  ), data=dat13.4r,chains=4,log_lik=TRUE)  precis(m13.4r,depth=2)  plot(precis(m13.4r,depth=2))  pairs(m13.4r, omit ="cutpoints")  traceplot(m13.4r)  trankplot(m13.4r)  # detect the number of cores of your CPU  options(mc.cores = parallel::detectCores())  ### Limit ~ ExploitFlower##  dat13.5r <-list(  O =as.integer(response),  EF =as.integer(exploit_flower_s),  R =as.integer(Region))  m13.5r <- ulam(  alist(  O ~dnorm(EF,p),  logit(p) <-a[R],  a[R] ~dnorm(0,1.5)  ), data=dat13.5r,chains=4,log_lik=TRUE)  precis(m13.5r,depth=2)  plot(precis(m13.5r,depth=2))  pairs(m13.5r, omit ="cutpoints")  traceplot(m13.5r)  trankplot(m13.5r)  ###Visualization###  compare(m13.1e,m13.2e,m13.3e,m13.4e,m13.5e)  plot(compare(m13.1e,m13.2e,m13.3e,m13.4e,m13.5e))  compare(m13.1r,m13.2r,m13.3r,m13.4r,m13.5r)  plot(compare(m13.1r,m13.2r,m13.3r,m13.4r,m13.5r))  compare(m13.1e,m13.2e,m13.3e,m13.4e,m13.5e,m13.1r,m13.2r,m13.3r,m13.4r,m13.5r)  plot(compare(m13.1e,m13.2e,m13.3e,m13.4e,m13.5e,m13.1r,m13.2r,m13.3r,m13.4r,m13.5r)) |
| --- |
